# Conducting public involvement in dementia research: The contribution of the European Working Group of People with Dementia to the ROADMAP project

**DOI:** 10.1111/hex.13246

**Published:** 2021-04-06

**Authors:** Ana Diaz, Dianne Gove, Mia Nelson, Michael Smith, Claire Tochel, Christophe Bintener, Amanda Ly, Christin Bexelius, Anders Gustavsson, Jean Georges, John Gallacher, Cathie Sudlow

**Affiliations:** ^1^ Alzheimer Europe Luxembourg City Luxembourg; ^2^ Centre for Medical Informatics Usher Institute of Population Health Sciences and Informatics University of Edinburgh Edinburgh UK; ^3^ F. Hoffmann‐La Roche Ltd. Basel Switzerland; ^4^ Quantify Research Stockholm Sweden; ^5^ Dementias Platform UK University of Oxford Oxford UK

**Keywords:** Alzheimer's disease, dementia, patient empowerment, patient engagement, patient involvement, public participation

## Abstract

**Background:**

Dementia outcomes include memory loss, language impairment, reduced quality of life and personality changes. Research suggests that outcomes selected for dementia clinical trials might not be the most important to people affected.

**Objective:**

One of the goals of the ‘Real world Outcomes across the Alzheimer's Disease spectrum for better care: Multi‐modal data Access Platform’ (ROADMAP) project was to identify important outcomes from the perspective of people with dementia and their caregivers. We review how ROADMAP's Public Involvement shaped the programme, impacted the research process and gave voice to people affected by dementia.

**Design:**

The European Working Group of People with Dementia (EWGPWD) were invited to participate. In‐person consultations were held with people with dementia and caregivers, with advance information provided on ROADMAP activities. Constructive criticism of survey content, layout and accessibility was sought, as were views and perspectives on terminology and key concepts around disease progression.

**Results:**

The working group provided significant improvements to survey accessibility and acceptability. They promoted better understanding of concepts around disease progression and how researchers might approach measuring and interpreting findings. They effectively expressed difficult concepts through real‐world examples.

**Conclusions:**

The role of the EWGPWD in ROADMAP was crucial, and its impact was highly influential. Involvement from the design stage helped shape the ethos of the programme and ultimately its meaningfulness.

**Public contribution:**

People with dementia and their carers were involved through structured consultations and invited to provide feedback on project materials, methods and insight into terminology and relevant concepts.

## 1 INTRODUCTION

Alzheimer's disease is the most common cause of dementia, accounting for 50%‐70% of cases.^1^, ^2^ It is associated with a range of outcomes, including memory loss, language impairment, increased dependency, reduced quality of life and personality changes.^3^, ^4^ Research has suggested that outcomes selected for clinical trials in dementia might not be important to people with the condition and their caregivers.^3^, ^4^, ^5^, ^6^ Selecting outcomes of importance to key stakeholders reportedly improves the relevance and quality of research results, makes studies more acceptable to potential participants and increases participant recruitment and retention.^7^, ^8^, ^9^

One of the goals of the ‘Real world Outcomes across the Alzheimer's Disease spectrum for better care: Multi‐modal data Access Platform’ (ROADMAP) project^10^ was to identify the outcomes of Alzheimer's disease that are most important to people with the disease and their caregivers, understand what would constitute a meaningful delay in disease progression from their perspectives and create a disease progression matrix that highlights the outcomes of greatest importance at the various stages of the disease (i.e. from pre‐clinical Alzheimer's disease to Alzheimer's dementia). Findings would inform the development of a data platform that integrates multiple sources of real‐world data related to Alzheimer's disease, called the Data Cube,^11^ to enhance researchers' understanding of disease progression in the real world. To achieve this goal, ROADMAP involved people with dementia and caregivers in the research process, not only as participants but also as advisors. This is in keeping with the concept of ‘Public Involvement’ (PI) (formerly known as Public and Patient Involvement [PPI]).^12^

PI is not a specific method, but an approach to involving people in research other than as research participants. It is about conducting research with and by people with a particular condition and/or members of the public, rather than research being done to, for or about them.^13^, ^14^, ^15^, ^16^ PI can be seen as a ‘means to an end’, in the sense of attempting to improve the quality, relevance and utility of research (from both research and user perspective), but also as ‘an end in itself’, in the sense of being linked to democracy (e.g. democratic decision making, public accountability, legitimization and transparency), people having rights (e.g. a right to voice, a right to be involved in research relevant to one's own condition) and ethical principles of justice and fairness.^12^ Involvement can occur at all stages of research, including prioritization of studies,^17^, ^18^ design and management of research,^19^ identification of health outcomes,^20^, ^21^ data collection and analysis^22^, ^23^ and dissemination of findings.^24^

Over the last few decades, there has been an increased interest in involving people living with dementia in PI in dementia research.^25^ Several national and European‐funded research projects have developed different approaches and methodologies to achieve this,^25^, ^26^, ^27^ although information regarding the impact of PI activities for researchers, the people involved and society is still lacking. From the perspective of people affected by the condition, PI in dementia research is considered as fundamental and should become the norm, but efforts still need to be made to ensure PI is inclusive of a diverse set of people.^28^

### 1.1 Aims

This article reviews how ROADMAP's PI consultations

1. shaped the design and content of the stakeholder surveys,
2. had an impact on other issues related to the research process in general and
3. gave voice to people with dementia and caregivers.

We believe it is a success story for meaningful and active involvement of people with dementia and their caregivers in the research process.

## 2 ORGANIZATION OF PUBLIC INVOLVEMENT IN ROADMAP

### 2.1 Selection of the people involved

#### 2.1.1 People with dementia

People with dementia from the European Working Group of People with dementia (EWGPWD) took part in this consultation. The EWGPWD is composed of people from different countries and was set up by Alzheimer Europe in 2012.^29^ The members of the group had all been nominated by their national Alzheimer Associations. They work in collaboration with Alzheimer Europe, contributing towards Alzheimer Europe's work and research projects in which it is involved. The group works to ensure that the activities, projects and meetings of Alzheimer Europe duly reflect the priorities and views of people with dementia. All members of the EWGPWD are in the mild or moderate stages of dementia and have capacity to understand what is being asked of them. Alzheimer Europe was a member of the ROADMAP consortium, and the EWGPWD agreed to be involved in this consultation activity as part of their PI remit. Overall, nine people with dementia (eight female and one male) from the Czech Republic, Germany, Portugal, the Republic of Ireland, Sweden and the United Kingdom were involved in the consultations.

#### 2.1.2 Informal caregivers

Members of the EWGPWD are typically accompanied to meetings by a relative, friend and occasionally by a member of staff from an Alzheimer Association. However, on this occasion, the accompanying individuals were invited to be involved in a separate consultation for caregivers. Overall, seven caregivers (five female and two male) from Portugal, the Republic of Ireland, Sweden and the United Kingdom were involved in the consultations.

### 2.2 Planning and conducting the public involvement consultations

Consultations with the EWGPWD and their caregivers were held in Luxembourg on the 4th and 5th of September 2017 and were conducted in accordance with Alzheimer Europe's position on PI,^16^ which was developed in collaboration with the EWGPWD and the INTERDEM research groups.^30^ Alzheimer Europe has great experience in preparing and conducting PI work with people with dementia. The schedule was developed by Alzheimer Europe in collaboration with ROADMAP members from the University of Edinburgh. In addition, the Dementia Engagement and Empowerment Project (DEEP) guidelines^31^ were taken into account when developing the materials and the schedule.

Involvement consisted of two concurrent consultations (one for the members of the EWGPWD and the other for their caregivers) that ran over the course of 2 days. Consultations were conducted in English, which is standard practice for the EWGPWD. All people involved received information about the project and the topics to be discussed 2 weeks prior to the consultation. In addition, on the first day of the consultation, a presentation about ROADMAP, relevant concepts in the project (e.g. real‐word evidence and outcomes in research) and why PI was important in the project, was provided.

The consultations were organized in such a way as to ensure that the EWGPWD and their caregivers were actively involved in the design and interpretation of ROADMAP's research activities. They facilitated productive interaction between the research team and stakeholders and informed and guided the translation of the results. The key discussion topics aimed

1. to provide constructive criticism of the stakeholder surveys, relating to its content, layout and accessibility and
2. to share views and perspectives on
  - their personal experiences of disease progression and the outcomes that typically indicate the disease is progressing,
  - staging disease progression and
  - what would constitute a meaningful delay in disease progression.

The consultations were jointly planned and carried out by members of ROADMAP from the University of Edinburgh and staff of Alzheimer Europe with expertise in PI in dementia. Alzheimer Europe's position paper on PI in dementia research highlights the different issues that need to be carefully considered in PI, including planning the involvement, establishing roles and responsibilities, providing the necessary support, managing information and input from PI, recognizing the contribution of people with dementia involved in research in this way, promoting and protecting the rights and well‐being of people with dementia and promoting an inclusive approach and the necessary infrastructure for PI in dementia research.^16^ Reflecting on and addressing these issues was key for the quality of the PI activities. These principles were addressed in the consultations and a number of factors were identified which improved their quality:

1. Having a clear introduction to the topic of interest, written without jargon and giving a detailed lay explanation of the issue.
2. Providing this information in writing in advance: at least 2 weeks in advance of the activity.
3. Being precise about what is wanted from the group and from their involvement in the project.
4. Explaining the topic of interest again on the day and giving time to answer any questions or explain the issue in further detail as required.
5. Limiting the activities to a set time and taking adequate breaks between each consultation activity.
6. Ensuring people talk one at a time and avoiding individuals talking over each other.
7. Allowing time (gaps in the conversations) for the members to reflect on and add to the discussion when they are ready.

### 2.3 Synthesis of discussions

With the authorization of all members of the EWGPWD and their caregivers, all consultation activities were audio‐recorded and notes were made. This enabled the moderators of the consultations to be fully involved in the discussions and to be attentive to possible needs of members of the EWGPWD (e.g. signs of fatigue, irritation or difficulties understanding). After the consultation, recordings were transcribed verbatim. The notes taken during the meeting and photographs of the different activities (e.g. flipchart summaries and prioritization activity) were also used for the analysis.

A thematic analysis approach was taken to organize the input provided during the consultations in a systematic and meaningful way. This involved the researchers from Edinburgh University reading the transcripts repeatedly and grouping the different perspectives and ideas into possible topics, for example what outcomes are important and why are specific outcomes important. This was reviewed and discussed with the other facilitators involved (Alzheimer Europe) and later with the people involved in the PI activity.

The report was presented to the EWGPWD on 27 June 2018 in Brussels to confirm that their input had been represented accurately. Following this, findings were reported to ROADMAP's Expert Advisory Group^32^ on 29 June 2018 in Amsterdam, to discuss the relevance of the group's input and address any gaps. The subsequent findings and the conclusions drawn were agreed by all parties.

## 3 IMPACT OF PUBLIC INVOLVEMENT ON THE ROADMAP STUDY

### 3.1 Survey refinement

The survey had been drafted with the intention that it would be completed online and in paper by people with dementia, their caregivers and health‐care professionals throughout Europe. It included a list of outcomes related to Alzheimer's disease, with the intention of asking participants to rate how important each outcome was with regard to assessing a meaningful delay in disease progression, in addition to questions relating to disease stage, caregiving duties and demographics.^33^

The EWGPWD provided constructive criticism of the survey design and content. An important issue addressed by the members of the EWGPWD and caregivers was related to the terminology used to refer to Alzheimer's disease in the survey. Important conceptual changes were made in this regard based on the feedback provided in the consultation. The original survey used the terms Alzheimer's disease and dementia. Members of the EWGPWD felt this was confusing and that many people with dementia and caregivers would not be familiar with the difference between both terms (i.e. new conceptualization of Alzheimer disease including pre‐dementia as well as dementia stages). It was therefore suggested to use the term dementia and, if necessary, to separately refer to the pre‐dementia stages. It was also advocated to include people with any type of dementia in the study and not just Alzheimer's as often; in ‘real life’, people are not aware of their differential diagnosis, or their diagnosis is mixed (e.g. Alzheimer's and another type of dementia). Another important concern was in relation to the accessibility of the survey. Their suggestions have been compiled into a list of ‘dos’ and ‘don'ts’ with regard to developing surveys for people with dementia, shown in Table 1.

**TABLE 1:** Survey refinements suggested by the EWGPWD

Category	Do	Don't
Format and style	Include questions with check boxes or yes/no answers, as they are easier to comprehend	Use a small or ineligible font size
	Use bullet points, colour and imagery where possible	Use bold or italicised text
Language: clarity	Consider how questions are asked e.g. ask for date of birth rather than age, as this is easier to remember	Use words with multiple meanings or homonyms
Language: tone	Make questions short, simple and direct	Make questions passive, repetitive or patronising
Support and ease of access	Carefully consider survey distribution methods, e.g. do not leave surveys in a memory clinic reception as these will likely be ignored	
	If administered by a professional, make sure they are adequately trained and understand living with dementia	
	Use multiple survey mediums e.g. paper and electronic	
Effort and focus required	Provide a clear indication of how long the survey will take to complete	Include questions that require a large amount of writing or typing, e.g. ‘please expand or add more information about your choice’
	Make the survey short	
	Keep in mind participants' fatigue or stress when developing questions	

Input from PI was used to clarify and simplify some terms and questions in the survey which was ultimately perceived as more accessible and clear by the people with dementia and caregivers involved in the PI activity. Following the feedback provided in the PI consultation, the survey was completed by 456 people, including 74 people with dementia, 144 caregivers and 238 health‐care professionals.^33^

### 3.2 Enhancing our understanding of key concepts

The consultations provided relevant input for the ROADMAP project regarding the topics of important outcomes, disease progression, staging of dementia and meaningful delay in dementia progression.

#### 3.2.1 Meaningful outcomes

An important goal of the ROADMAP project was to identify outcomes that are important to people with dementia and their caregivers. In the context of the PI consultation, the EWGPWD and their caregivers raised a number of outcomes that they considered important for assessing disease progression, or that would constitute a meaningful delay in disease progression if preserved. A total of 38 outcomes were identified, and these were organized into eight overarching domains, namely cognitive abilities, functional abilities, behavioural and neuropsychiatric symptoms, quality of life of the person with dementia, impact on carers, medical investigations, mortality and comorbidities, and significant disease‐related life events. These outcomes have been integrated into key ROADMAP outputs.^11^, ^33^

#### 3.2.2 Disease progression

Progression can be a sensitive and difficult topic. Nevertheless, as for any other medical condition, understanding the progression of dementia is very important for people affected by the condition. Progression was described as a complex issue, and in particular, people with dementia highlighted the need to consider how the experience of multiple symptoms can influence disease progression. For example, individual symptoms, when combined, may influence the perception of disease progression. They also explained that people with dementia could sometimes be misunderstood because of behavioural or communication problems and may experience unusual emotional responses in ‘normal’ circumstances. They explained that together, these could lead to relationship problems and isolation, which impacted their quality of life and their perception of the severity of their dementia.

It was recommended that the researchers should consider the following series of issues when thinking about disease progression.

##### Assessing and measuring progression

Overall, the groups were of the opinion that brain scans can be useful for confirming disease progression and diagnosis, but they currently hold little meaning in terms of experience of symptoms in everyday life, hence lacking tangible benefit. Overall, this lack of connection between the experience of symptoms and the results of scans was considered to limit their usefulness when identifying disease progression.

In relation to measurement scales and tests (e.g. pen and paper tests), they highlighted that completing these tests can be disheartening for some people (like ‘…watching yourself decline’). This, it was suggested, can lead to a loss of confidence, whilst offering little clarity with regard to disease progression. They also raised the issue that such scales and tests often focus solely on cognitive measurements, with other important outcomes being overlooked, that is assessing cognition in the absence of everyday functioning.

I have a test every six months… And I've actually just told them that I'm not doing it anymore, because every test I've done every six months it's never even, I'm not asking for miracles, but it's never even been the same. I always lose a point or a couple of points, so I've said right, I don't want to lose any more confidence, so I'm not bothered now. I know it's progressing, yeah. (EWGPWD member)

##### Personalization and building the individual's story

People with dementia and caregivers suggested that assessing progression should be personalized, as everyone's story is different and there is huge variability in symptom experience. Caregivers suggested that living with the person with dementia is important for assessing and understanding progression, as new and recurrent behaviours can be observed. This allows for differentiation between true progression and simply having a ‘bad day’.

##### Taking a holistic perspective of people with dementia

It was highlighted that when looking at the progression of dementia, other comorbidities and impairments should be taken into account and, in particular, the potential impact of these on the progression or the perceived progression of the disease. This is important as they can directly influence assessment, for example poorer cognitive or functional performance due to a comorbidity, as opposed to dementia.

#### 3.2.3 Staging dementia

It was generally perceived that existing scales to stage dementia do not always capture the individual's lived experience. However, there was a general consensus that the terms ‘mild – moderate – severe’ adequately captured the stages of the condition in a way that could be easily understood by people with dementia, as they provide a good approximation of how far a person's dementia has progressed.

A number of issues were raised in relation to staging disease progression, which should be considered by researchers, discussed in turn.

##### Inconsistency and susceptibility to external influences

Some people felt that judgement of a person's stage can differ between clinicians, leading to inconsistencies. Further, assessing stage using clinical measures only provides a snapshot of the condition, which can be heavily influenced by factors like mood and the environment in which the assessment took place. It was concluded that the assessment of stages should therefore be tailored to the individual, given differences in the type of symptoms experienced and rate of progression.

##### Implications for daily life

The groups pointed out that staging dementia has implications for everyday life, such as the loss of a driving licence, self‐determination, freedom of movement and management of finances. In some countries, restrictions can occur simply due to a diagnosis of dementia, without assessment of capacity or skills. Inversely, a few people reported that diagnosis and the staging of dementia can lead to gaining rights or access to services in some countries that would otherwise be unavailable.

My belief is we require labelling for social work professionals, in some people in day‐to‐day life, having labels is irrelevant, it's irrelevant for my dad. What is relevant is I needed an explanation for why [he] couldn't function and work in his 50s. (Caregiver)

##### Preferences for new staging frameworks

The EWGPWD and caregivers stated that new staging frameworks should account for fluctuations or variability in a person's condition, as current frameworks can be rigid and linear. This could be achieved by providing more flexibility between the boundaries of the stages.

The EWGPWD's feedback on the conceptualization of stages was fed into the survey design^33^ and ROADMAP's Data Cube^11^ as it was considered useful and meaningful to people with dementia and their caregivers.

#### 3.2.4 Meaningful delay

Three relevant, interconnected issues were addressed in this context, namely delay before the onset of dementia, delaying the progression of dementia and the balance between longevity and quality of life.

##### Delaying the onset of dementia

It was felt that meaningful delay is a complex concept, which might not be clearly understood by some people. It would be important to clearly distinguish between cure, prevention and meaningful delay. Meaningful delay was described in positive terms and often linked to the idea of ‘enjoying or having some extra time’. Important issues when talking about a meaningful delay of the onset of dementia were related to the certainty that the person would eventually develop dementia, how long such delay would be, the balance between potential risks and benefits (e.g. side‐effects, having a faster deterioration after the delay, better quality of life/independence) and the particular circumstances of each person (e.g. comorbidities, age, frailty). It was highlighted that the meaning of the delay may also depend on whether the person is or is not familiar with dementia.

Okay. It's to delay the onset. Onset to me means the start of dementia. Well I don't want to start having dementia, so give me the drug. But how do you know I'm going to get dementia? So to delay the onset, yeah give me the drug. [Laughs] But do I need it? (Caregiver)

Two members of the EWGPWD elaborated further.

A: If you haven't got dementia and know nothing about it then a drug that just delays it wouldn't have a big meaning, ‘if I'm going to get it why not just have it’…but if you know a lot about dementia then it would mean a big difference.

B: Yeah, because you know how it is, actually living with dementia…

A: You know how important it is… Yeah, how important an extra year or an extra month…

(EWGPWD members)

##### Delaying disease progression

The delay of disease progression was perceived as potentially having a bigger, more meaningful impact at the earliest stage of the condition. It was highlighted that delaying progression should be accompanied by experiencing a reduction of symptoms and improvement in the quality of life of the person. Delaying the progression in the advanced stages of the disease may be less helpful and arguably cruel. Again, balancing risk and benefit with regard to side‐effects was seen as hugely important.

##### Longevity vs quality of life

The importance of quality of life was raised, with a consensus that there is little benefit in delaying progression and prolonging life if there is no benefit in terms of quality of life (i.e. maintaining or improving quality of life). Longevity was not deemed an important measure in its own right. Being able to guarantee a person with dementia additional ‘good years’ was considered the greatest value.

I don't want it to happen but if it's going to happen, let's get on with it. That's why, if it can't be guaranteed, a good ten years…. (Caregiver)

Whilst some common themes were detected in relation to the concept of meaningful delay, the groups agreed that an individual's own set of values was of priority when determining choices related to their life and treatment and believed that respect for such individual differences was paramount.

### 3.3 Democracy, rights and justice

The EWGPWD and caregivers were keen to have their voices heard in the ROADMAP study (in the sense of having a ‘right to voice’), as well as sharing their experiences and perspectives based on living with dementia with the ROADMAP researchers. This was seen as involving mutual respect and being based on the principle of reciprocity. Table 2 provides a summary of feedback provided by members of the EWGPWD when asked to reflect on the PI process and to identify the most important messages they wished to pass on to ROADMAP researchers.^34^

**TABLE 2:** Reflections on ROADMAP's PI consultations from the EWGPWD and their caregivers

Impact level	Reflections from the EWGPWD and their caregivers
Researchers	It was important to educate researchers about what it is like to live with dementia, to look at people with dementia as human beings and understand what their condition is like. The EWGPWD and their caregivers found sharing their experiences enriching
	There is no need to rewind the clock. Researchers should try to collaborate more
People with dementia and carers	Providing feedback was important to the EWGPWD and their caregivers, as researchers often do not do this
	Everyone with dementia has a contribution to make, regardless of their education level or background
	Involving people with dementia in PI can change perceptions of what it is like to be involved in dementia research. The EWGPWD could return to their home countries and talk about their experiences of being involved in ROADMAP's PI consultations
	Being involved in the ROADMAP consultations gave the EWGPWD confidence and hope and helped them better understand their own conditions
Research	People with dementia and caregivers should be involved as early as possible and should continue to be involved throughout the research process
	The researchers maintained a professional attitude, but never lost the human touch. This was viewed as very important to the consultations' success
	Take feedback and advice from PI forward and incorporate it into the research, do not leave it to gather dust

### 3.4 Key outputs and deliverables

The input of the EWGPWD and their caregivers has been incorporated into a number of key ROADMAP outputs, including a deliverable reporting stakeholder‐generated lists of real‐world evidence–related outcomes of Alzheimer's disease across the spectrum,^33^ a disease progression and outcome classification matrix^33^ that fed into the development of ROADMAP's Data Cube,^11^, ^35^ a presentation at the Alzheimer's Disease International Conference 2018 ‘Women and Dementia’ session,^36^ presentations at the Alzheimer Europe 2018 Conference ‘Real‐World Evidence in Alzheimer's Disease’ session,^37^ refinement of ROADMAP's stakeholder survey, which was subsequently completed by 456 individuals,^33^ and the present article.

Additionally, ROADMAP's Data Cube is being promoted and developed further in collaboration with the Neuronet project,^38^ a collaborative effort aimed at bringing together the findings and learnings of 15 Innovative Medicines Initiative funded projects relating to neurodegeneration. The input of the EWGPWD in ROADMAP has had a lasting impact in the field of Alzheimer's disease and dementia.

## 4 CONCLUSION

This paper presents an example of people affected by dementia involved in PI activities in a particular European project and what impact this involvement had on the ROADMAP project. The Alzheimer Europe's position paper^16^ highlights the importance of involving people affected by dementia from the very beginning and for their involvement to be continued until the end of the research project. In this project, people affected by dementia were involved in a 2‐day consultation and at the end of the project. In addition, one person with dementia participated in the project's Expert Advisory Group.^32^ The involvement of people affected by dementia could have been even more impactful had they been involved earlier on and throughout the project.

Nevertheless, the views and reflections of people directly affected by the condition had a strong impact on survey design and on the development and use of concepts throughout the whole project. People affected by dementia appreciated and enjoyed being involved in this way in the project. In addition, the discussions helped the researchers to understand better the perspectives of people affected by dementia with regard to issues that were important for the project, such as meaningful outcomes, progression and delay of the disease, and also to identify issues and concerns that should be taken into account when carrying out research. The progression and potential delay of dementia are very important and sensitive topics for people affected by dementia. Assessing the progression of the disease is important and necessary, but the potential impact that this may have on a person's well‐being and quality of life also needs to be taken into account and addressed. The potential delay of the disease can give people hope, but it is also important that people understand clearly what it entails and its potential limitations. The individual experience and considering the person as a whole (including comorbidities, social and personal factors) were key messages for ROADMAP researchers working on these topics.

Involvement of people with dementia and caregivers in research through PI is paramount, but requires careful consideration of how to plan and conduct it. This includes, amongst other issues, ensuring that relevant expertise, time and budget are available.

## CONFLICT OF INTEREST

AG was a partner of Quantify Research, providing consultancy services to pharmaceutical companies and other private and public organizations and institutions. AG's contribution to ROADMAP was on behalf of Roche Pharmaceuticals. C Bintener received an honoraria from ProLog Wissen GmbH, Cologne, Germany, for a cognitive training programme.

## AUTHOR CONTRIBUTIONS

Ana Diaz and Dianne Gove designed the study, carried out consultative work, analysed data and shaped and drafted the manuscript. Mia Nelson designed the study, carried out consultative work, analysed data and contributed to the manuscript. Michael Smith analysed data and shaped and drafted the manuscript. Claire Tochel reviewed analysis and shaped and drafted the manuscript. Christophe Bintener and Amanda Ly designed the study, carried out consultative work and reviewed the manuscript. Christin Bexelius, Anders Gustavsson, Jean Georges, John Gallacher and Cathie Sudlow designed the study and reviewed the manuscript.

## Data Availability

Data sharing is not applicable to this article as no new data were created or analysed in this study.
